# Quantitative and Functional Assessment of the Influence of Routinely Used Cryopreservation Media on Mononuclear Leukocytes for Medical Research

**DOI:** 10.3390/ijms23031881

**Published:** 2022-02-07

**Authors:** Patrick Haider, Timothy Hoberstorfer, Manuel Salzmann, Michael B. Fischer, Walter S. Speidl, Johann Wojta, Philipp J. Hohensinner

**Affiliations:** 1 Department of Internal Medicine II, Division of Cardiology, Medical University of Vienna, 1090 Vienna, Austria; patrick.haider@meduniwien.ac.at (P.H.); n11700883@students.meduniwien.ac.at (T.H.); manuel.salzmann@meduniwien.ac.at (M.S.); walter.speidl@meduniwien.ac.at (W.S.S.); 2 Ludwig Boltzmann Institute for Cardiovascular Research, Medical University of Vienna, 1090 Vienna, Austria; philipp.hohensinner@meduniwien.ac.at; 3 Department of Blood Group Serology and Transfusion Medicine, Medical University of Vienna, 1090 Vienna, Austria; michael.b.fischer@meduniwien.ac.at; 4 Core Facilities, Medical University of Vienna, 1090 Vienna, Austria; 5 Center for Biomedical Research, Medical University of Vienna, 1090 Vienna, Austria

**Keywords:** clinical studies, cryopreservation, MNCs, monocyte subsets, lymphocytes

## Abstract

Quantitative and functional analysis of mononuclear leukocyte populations is an invaluable tool to understand the role of the immune system in the pathogenesis of a disease. Cryopreservation of mononuclear cells (MNCs) is routinely used to guarantee similar experimental conditions. Immune cells react differently to cryopreservation, and populations and functions of immune cells change during the process of freeze–thawing. To allow for a setup that preserves cell number and function optimally, we tested four different cryopreservation media. MNCs from 15 human individuals were analyzed. Before freezing and after thawing, the distribution of leukocytes was quantified by flow cytometry. Cultured cells were stimulated using lipopolysaccharide, and their immune response was quantified by flow cytometry, quantitative polymerase chain reaction (qPCR), and enzyme-linked immunosorbent assay (ELISA). Ultimately, the performance of the cryopreservation media was ranked. Cell recovery and viability were different between the media. Cryopreservation led to changes in the relative number of monocytes, T cells, B cells, and their subsets. The inflammatory response of MNCs was altered by cryopreservation, enhancing the basal production of inflammatory cytokines. Different cryopreservation media induce biases, which needs to be considered when designing a study relying on cryopreservation. Here, we provide an overview of four different cryopreservation media for choosing the optimal medium for a specific task.

## 1. Introduction

The analysis of human mononuclear cells (MNCs), which comprise circulating monocytes and lymphocytes, has become an invaluable tool in medical research and nowadays even in experimental clinical cell-therapies including autologous dendritic cell and chimeric antigen receptor T (CAR-T) cell immunotherapy [1,2,3,4]. Further, the absolute amounts and the relative composition of individual leukocyte populations can be used as reliable biomarkers and predictors in different (inflammatory) diseases, i.e., an increase of circulating CD14^++^CD16^+^CCR2^+^ intermediate monocytes (Mon2) was found to be independently predictive for cardiovascular events or outcome in multiple cardiovascular diseases [5,6,7,8]. Functional immunological assessment of MNCs ex vivo has further important implications in basic [9] as well as in therapeutic immunological research, as has been shown, for example, in the assessment of vaccine-induced cell-based immune responses during the COVID-19 pandemic [10,11].

Assessment of MNCs in research laboratories in the context of clinical trials requires personal and technical resources, i.e., instruments and standardized protocols for flow cytometry [12] and the respective technicians processing and analyzing the samples in an appropriate time. Especially in multi-centric studies, not all centers perform the laboratory analysis of their samples themselves. These pre-processing and pre-analytic steps challenge a standardized analysis, which is required to draw solid scientific conclusions [13]. Cryopreservation of MNCs has become an important method to overcome these issues, allowing transportation and batch-wise analysis of cells, which reduces inter-laboratory and intra-assay biases [14,15]. However, cryopreservation induces various quantitative and functional alterations in different immune cell populations, which needs to be considered when studies using cryopreserved MNCs are interpreted [16,17,18,19]. Due to these observations, the European Society of Cardiology recommends the use of only fresh samples for monocyte subset analysis [20].

In this study, we aimed to compare the effects of four routinely used cryopreservation media [CryoStor CS10 (Stemcell, Vancouver, Canada); Synth-a-Freeze (ThermoFisher, Waltham, MA, USA); 90% FBS + 10% DMSO (self-prepared); 70% RPMI1640 + 20% FBS + 10% DMSO (self-prepared)] on the recovery/viability and the amount/function of different leukocyte populations in detail. Therefore, leukocyte populations were quantified by flow cytometry before cryopreservation as well as directly after and after 1-h resting period at 37°C post thawing. Further, the immunological response of the MNCs was assessed by qPCR, ELISA, and flow cytometry of cytokines and activation markers.

The choice of used cryopreservation medium affected the obtained recovery and viability after cryopreservation. Further, we were able to verify previously reported findings on relative alterations in T cell and monocyte subsets. Freeze–thawing increased the basal production of pro-inflammatory activation markers and cytokines in all cryopreservation media. By stimulating the toll-like receptor 4 (TLR4) -competent cell compartment of MNCs with lipopolysaccharide, we were able to show that the used cryopreservation medium changed the immune response significantly. Further, the expression of activated CD11b on the surface of monocytes was affected by the used cryopreservation medium. To summarize our findings, we ranked the different cryopreservation media based on the differences in conditions before freezing and the observed standard deviation for each experimental aspect and created an overview table to aid other research groups in selecting an appropriate cryopreservation medium for their future studies.

## 2. Results

### 2.1. The Choice of Cryopreservation Medium Affects Recovery and Viability of MNCs after Thawing

**Figure 1:**
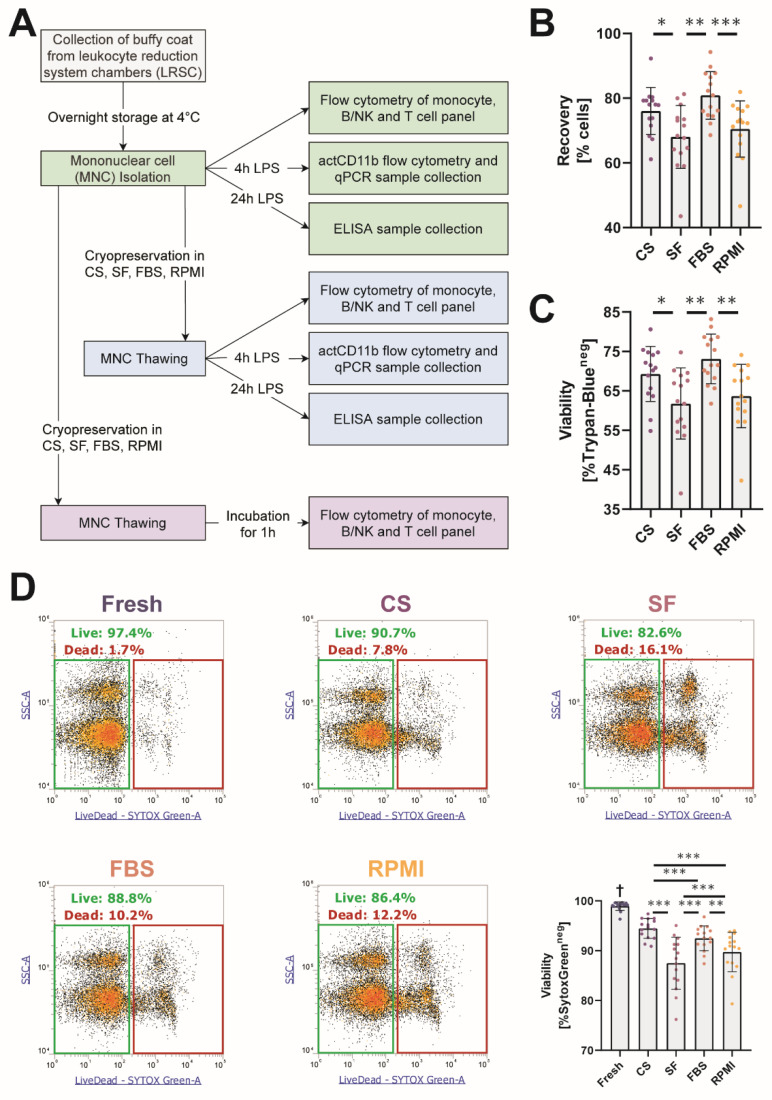
The choice of cryopreservation medium affects recovery and viability of mononuclear cells (MNCs) after thawing. (**A**) Experimental workflow of this study. (**B**) Calculated recovery after thawing of total cells frozen; 1 × 10^7^ cells were frozen per aliquot, and recovered MNCs were counted on a LUNA automated cell counter. (**C**) Relative viability of all recovered MNCs after thawing using 0.4% trypan blue staining solution. The viability was analyzed on a LUNA automated cell counter. (**D**) Representative plots and data of the relative viability of MNCs analyzed by flow cytometry. Dying cells were identified by the membrane-impermeable DNA-intercalating dye SytoxGreen. * *p* < 0.05; ** *p* < 0.01; *** *p* < 0.001; † = significantly different from all other conditions. (*n* = 15 individual human donors).

Four cryopreservation media (CryoStor CS10™ = CS; Synth-a-Freeze™ = SF; 90%FBS/10%DMSO = FBS; 70%RPMI/20%FBS/10%DMSO = RPMI), which are routinely used in medical research studies, were examined for their effects on MNCs. MNCs were isolated from leukocyte reduction chambers, which were stored overnight at 4°C before isolation. Overnight storage did not alter the viability relative distribution and activatability (Figure S1). The experimental workflow for sample processing in this study is given in Figure 1A. To determine whether there were differences in recovery and viability between the individual cryopreservation media, MNCs were counted after thawing on a LUNA Automated Cell Counter using 0.4% Trypan Blue Solution to assess viability before they were further processed for flow cytometry and cell culture experiments. Recovery was higher when MNCs were stored in FBS (80.9% [74.8–87.6]) and CS (78.0% [71.5–79.3]) compared to RPMI (72.5% [65.9–76.7]) and SF (68.4% [63.1–73.9]) (Figure 1B).

FBS (71.5% [68.3–78.7]) and CS (70.1% [64.4–74.8])-stored MNCs also showed higher viability measured by Trypan Blue compared to ones stored in RPMI (63.7% [59.8–70.2]) or SF (62.4% [55.9–68.9]) (Figure 1C). As automated detection of Trypan Blue staining relies on good visual focus and evenly dispersed cells, we verified the viability data by flow cytometry using the cell-impermeable DNA-intercalating dye SytoxGreen. SytoxGreen stains only cells with impaired membrane integrity, typically found during cell death. As expected, cryopreservation significantly decreased the amount of viable cells compared to conditions before freezing (99.2% [98.9–99.5]). When we compared the individual cryopreservation media, CS (94.7% [92.6–96.4]) showed the best viability, closely followed by FBS (92.6% [91.3–94.3]) and RPMI (90.8% [87.6–92.7]). MNCs stored in SF (88.4% [84.1–91.3]) showed the lowest viability in this validation experiment (Figure 1D). Taken together, we found significant differences between the individual cryopreservation media. CS and FBS seem to be superior to RPMI and SF in terms of cell recovery and viability.

### 2.2. Cryopreservation Alters the Relative Distribution of Leukocyte Populations

**Figure 2:**
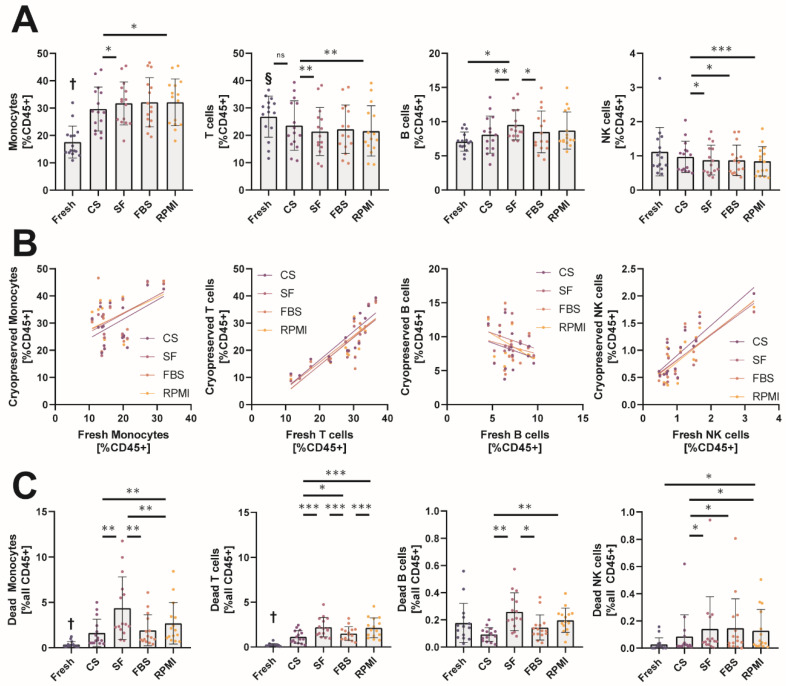
Cryopreservation alters the relative distribution of leukocyte populations. (**A**) Relative number of gated monocytes, T cells, B cells, and NK cells before and after cryopreservation analyzed by flow cytometry. Cell amounts are given as % live CD45+ cells. (**B**) Correlation plots of relative cell amounts of monocytes, T cells, B cells, and NK cells before and after cryopreservation. Correlation was analyzed by linear regression. (**C**) Analysis of dead monocytes, T cells, B cells, and NK cells before and after cryopreservation. Please notice the different scales on the y-axis of the two left and two right panels. Gated cells staining positive for intracellular SytoxGreen are shown relative to all CD45+ cells measured by flow cytometry. * *p* < 0.05; ** *p* < 0.01; *** *p* < 0.001; † = significantly different from all other conditions; § = significantly different from other conditions except the ones indicated with “ns”. (*n* = 15 individual human donors).

Next, we asked how the different cryopreservation media would affect the relative distribution of leukocyte populations after freeze–thawing, as it was shown before that cryopreservation can affect individual populations differently [21]. Therefore, MNCs were stained with three antibody panels and analyzed by flow cytometry to quantify the major leukocyte populations (gating is depicted in Figure S2). After cryopreservation, the relative number of monocytes and T cells compared to all CD45+ leukocytes, was drastically altered (Figure 2A). We observed increased amounts of monocytes after thawing in all used media. CS (28.5% [21.7–36.1])-stored MNCs resembled the distribution observed in fresh samples (14.5% [13.7–20.2]) better than MNCs stored in the other cryopreservation media: FBS (30.4% [25.4–38.7]), SF (32.5% [24.9–37.4]), and RPMI (34.1% [24.1–38.4]. The observed relative monocyte increase in general was due to a reduction in T cells after thawing. All cryopreservation media except CS (20.9% [16.8–31.8]) showed significantly lower amounts of T cells: FBS (19.9% [15.8–29.9]), SF (19.7% [15.7–26.5]), and RPMI (18.7% [15.4–28.2]) compared to the samples before freezing (28.5% [22.7–32.0]). B cell distribution was only significantly altered in samples stored in SF (8.7% [7.9–11.7]), whereas the other cryopreservation media CS (8.1% [6.1–10.5]), RPMI (7.3% [7.1–11.6]), and FBS (7.1% [6.1–10.5]) were not significantly different from fresh samples (7.1% [6.4–8.1]). Although NK cells were not significantly altered after thawing compared to fresh samples (0.9% [0.7–1.5]), we detected a significant difference between NK cells stored in CS (1.0% [0.6–1.2) compared to the other media, RPMI (0.8% [0.4–1.2]), FBS (0.8% [0.5–1.0]), and SF (0.7% [0.5–1.2]), with the median being closest to the fresh condition in CS-stored MNCs.

Next, we wanted to know whether the measured counts of the individual leukocyte populations would correlate before and after cryopreservation, which would be important for longitudinal studies comparing changes in individual patients rather than changes in the means of whole populations (Figure 2B). The individual linear regressions for each leukocyte population are given in Figure S3A. For monocytes, the overall correlation before and after freezing was very weak (R^2^ values: CS 0.2675, SF 0.2564, FBS 0.1760, RPMI 0.1633) and only significant for CS-stored monocytes. T cells (R^2^ values: CS 0.7589, SF 0.7543, FBS 0.6852, RPMI 0.7203) and NK cells (R² values: CS 0.7308, SF 0.5601, FBS 0.5890, RPMI 0.6731) correlated significantly in all cryopreservation media, with CS showing steeper linear regression lines than the other cryopreservation media. Importantly and unpredicted, B cell amounts before and after cryopreservation did not correlate at all in any medium. As recovery and viability were different between the cryopreservation media, we also assessed the absolute amount of dead cells for each leukocyte population. As shown in Figure 2B,C and NK cells were less affected by cryopreservation, with significant differences before and after cryopreservation only found for SF and RPMI. A significant number of monocytes and T cells underwent cell death after thawing compared to fresh samples (0.2% [0.13–0.4] for monocytes, 0.2% [0.1–0.2] for T cells), with higher amounts dying in SF (2.6% [2.0–6.5] for monocytes, 2.0% [1.4–3.2] for T cells) and RPMI (1.9% [1.1–3.4] for monocytes, 1.9% [1.2–3.2] for T cells) as compared to CS (0.8% [0.5–2.5] for monocytes, 0.9% [0.5–1.8] for T cells) and FBS (1.1% [0.8–3.2] for monocytes, 1.4% [0.8–2.0] for T cells). In summary, cryopreservation affected leukocyte populations differently, with some media performing better in maintaining the relative distribution of the MNC populations before and after freeze–thawing. Further, correlation before and after cryopreservation was very different between the individual leukocyte populations.

### 2.3. Analysis of the Effect of Cryopreservation Media on the Relative Frequency of Individual Leukocyte Subsets

**Figure 3:**
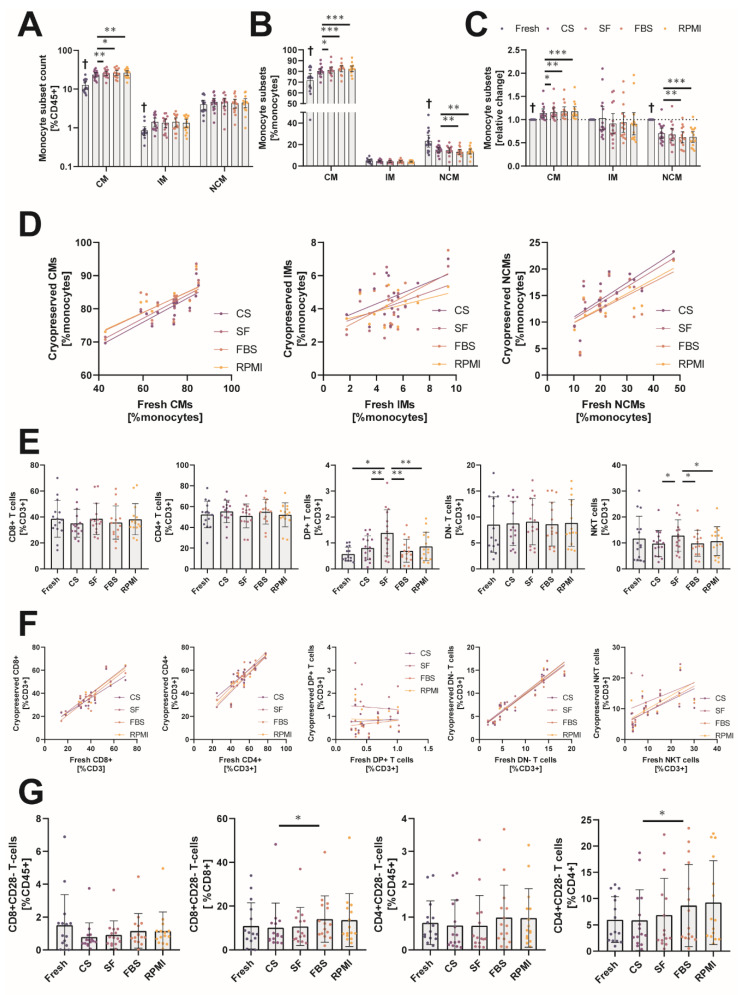
Analysis of the effect of cryopreservation media on the relative frequency of individual leukocyte subsets. (**A**) Relative amount of gated monocyte subsets relative to all live CD45+ cells analyzed by flow cytometry. CM = classical monocytes, IM = intermediate monocytes, NCM = non-classical monocytes. (**B**) Relative distribution of the individual monocyte subsets analyzed by flow cytometry. (**C**) Relative change in monocyte subset distribution before and after cryopreservation; >1 indicates a relative overrepresentation of a subset, <1 indicates a relative underrepresentation. (**D**) Correlation plots of relative monocyte subset distribution before and after cryopreservation. Correlation was analyzed by linear regression. (**E**) Relative amount of different T cell subsets relative to all live CD3+ T cells analyzed by flow cytometry. DP+ = double positive (CD4+, CD8+). DN- = double negative (CD4-, CD8-). (**F**) Correlation plots of different T cell subsets before and after cryopreservation. Correlation was analyzed by linear regression. (**G**) Relative amount of gated CD28- T cell subsets relative to all live CD45+ cells or to CD4/CD8+ cells analyzed by flow cytometry. * *p* < 0.05; ** *p* < 0.01; *** *p* < 0.001; † = significantly different from all other conditions. (*n* = 15 individual human donors).

Often, investigators are only interested in distinct leukocyte subsets such as CD4+ T cells in HIV [22] or relative subset distributions such as monocyte subsets in critically ill patients [23]. We analyzed the distribution of human monocyte subsets (classical CD14++CD16- = Mon1, intermediate CD14++CD16+ = Mon2 and non-classical CD14+CD16++ = Mon3) before and after cryopreservation. As shown above and similar to published data [16], the relative amount of monocytes in the CD45+ leukocyte compartment was significantly increased due to the death of other leukocyte populations. Interestingly, we were able to show that this increase was not evenly dispersed over all monocyte subsets. Cryopreservation led to a significant increase in total classical (Fresh 10.7% [8.8–17.2], CS 22.6% [19.0–28.8], SF 25.8% [21.9–31], FBS 25.2% [20.5–32.4], RPMI 28.0% [19.7–31.7]) and intermediate (Fresh 0.74% [0.68–0.90], CS 1.16% [0.90–2.17], SF 1.26% [0.71–1.78], FBS 1.15% [0.95–2.14], and RPMI 1.07% [0.87–1.98]) monocytes, whereas we did not observe an increase in non-classical (Fresh 2.9% [2.7–4.9], CS 4.4% [3.1–5.3], SF 4.6% [3.2–5.5], FBS 4.3% [2.9–5.1], and RPMI 4.6% [2.9–5.0]) monocytes (Figure 3A). When we compared the relative subset distribution, we found a significant increase in classical monocytes (Fresh 74.2% [64.1–81.2], CS 80.3% [78.1–81.8], SF 80.8% [78.5–82.8], FBS 83.4% [80.9–84.9], RPMI 82.3% [83.3–84.7]) whereas the proportion of non-classical monocytes (Fresh 20.1% [14.3–31.1], CS 15.6% [12.5–18.0], SF 15.4% [13.1–18.0], FBS 12.2% [11.1–17.7], RPMI 13.4% [11.7–15.8]) was significantly reduced (Figure 3B). By calculating the ratio of the relative change of the individual subset to the relative change of all monocytes overall, we were able to show that cryopreservation led to an overrepresentation of classical monocytes and a loss of non-classical ones (Figure 3C). These alterations were significantly less prominent in samples preserved in CS than in other cryopreservation media. We correlated the relative fraction of the individual subsets before and after cryopreservation. Only the classical (R^2^ values: CS 0.6651, SF 0.5826, FBS 0.4883, RPMI 0.5442) and non-classical (R^2^ values: CS 0.6030, SF 0.5035, FBS 0.3740, RPMI 0.4369) subsets were significantly correlated, whereas intermediate monocytes (R^2^ values: CS 0.2364, SF 0.1255, FBS 0.3208, RPMI 0.1440) did not correlate at all (Figure 3D). The individual linear regressions for each monocyte subset are given in Figure S3B.

Next, we focused on the different T cell subsets. We did not observe significant differences in the relative amount of CD8+ (Fresh 37.9% [30.8–43.9], CS 32.7% [29.0–40.7], SF 36.3% [30.7–42.2], FBS 35.4% [26.8–39.0], RPMI 35.4% [31.0–40.9]), CD4+ (Fresh 54.0% [43.0–61.0], CS 56.6% [49.7–61.5], SF 54.0% [45.8–57.7], FBS 56.7% [48.2–63.9], RPMI 54.3% [48.3–60.3]) or Double Negative (DN-) (Fresh 6.0% [4.4–14.0], CS 7.5% [5.0–13.8], SF 8.1% [4.3–13.6], FBS 7.2% [4.2–12.7], and RPMI 7.0% [4.8–13.1]) T cells. The amount of Double Positive (DP+) and Natural Killer-like T (NKT) cells was significantly increased after cryopreservation in samples stored in SF (1.2% [0.6–2.2] for DP+ and 12.0% [8.6–16.1] for NKT cells), whereas DP+ (FBS 0.6% [0.4–0.9], RPMI 0.7% [0.4–1.3] and CS 0.8% [0.4–1.1]) and NKT cells (FBS 8.8% [5.6–12.3], RPMI 11.9% [4.8–14.2] and CS 10.0% [4.9–12.5]) in the other cryopreservation media were comparable to fresh samples (0.5% [0.3–0.9] for DP+ and 9.1% [3.5–15.3] for NKT cells) (Figure 3E). These observations were also evident when we compared the correlations of the individual subsets. R² values were prominently lower for DP+ T cells and NKT cells than for the other subsets (Figure 3F). Loss of CD28 expression is associated with aging [24], and increased amounts of CD28- T cells were shown to be associated with the development of atrial fibrillation after cardiac surgery as well as with increased mortality in patients with known atrial fibrillation [25,26]. When we compared the effects of different cryopreservation media with the absolute amount and relative fraction of CD8+ and CD4+ T cells lacking CD28 expression (CD28-), we only observed small changes in the relative fraction of the CD4 and CD8 subsets in samples frozen in media containing FBS (11.6% [7.1–20.4] vs. 7.3% [4.1–18.6] for CD8+ and 4.4% [2.5–16.9] vs. 3.6% [2.0–10.4] for CD4+) (Figure 3G). These findings show that cryopreservation does affect individual leukocyte subsets to a varying degree, which needs to be considered when studies using cryopreserved leukocyte populations are compared to ones using fresh MNCs.

### 2.4. A 1-h Resting Period after Thawing Does Not Dramatically Alter Leukocyte Population Amounts

Lemieux et al. showed that a resting period of 1 h at 37°C in an incubator after cryopreservation could refine the expression of some surface markers, improving the relative distribution of leukocyte populations after thawing [19]. Therefore, we assessed a part of the cells after a 1-h resting period. However, in our experimental setup, we did not observe such prominent differences between unrested and rested MNCs.

**Figure 4:**
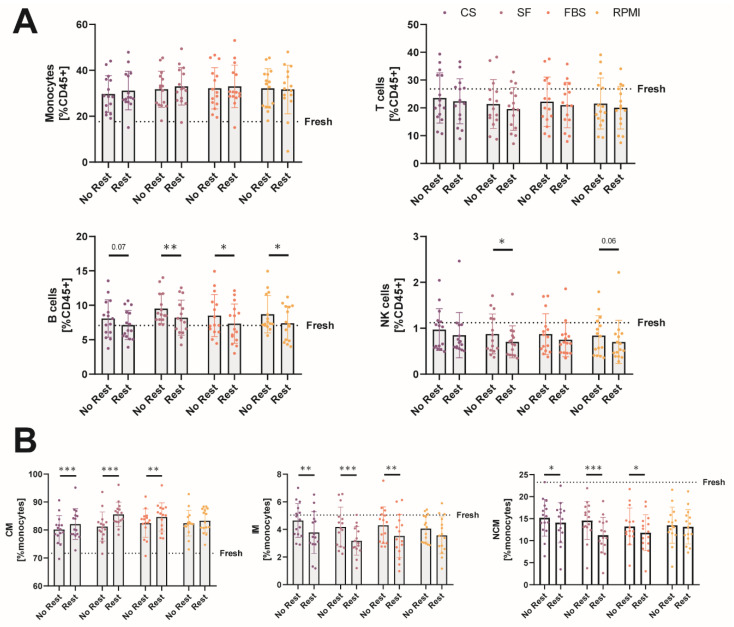
A 1-h resting period after thawing does not dramatically alter leukocyte population amounts. (**A**) Comparison of the relative amounts of monocytes, T cells, B cells, and NK cells without or with a 1-h resting period after cryopreservation before analysis using flow cytometry. The dotted lines labelled “Fresh” indicate the mean values of the fresh samples for each gated cell population. (**B**) Comparison of the relative distribution of monocyte subsets without or with a 1-h resting period after thawing before analysis using flow cytometry. The dotted lines labelled “Fresh” indicate the mean values of the fresh samples for each gated cell subset. * *p* < 0.05; ** *p* < 0.01; *** *p* < 0.001. (*n* = 15 individual human donors).

Compared to the analysis directly after thawing, B cell amounts were not significantly increased after a 1-h resting period (CS 8.1% [6.1–10.5] vs. 6.3% [5.5–9.0], SF 8.7% [7.9–11.7] vs. 7.8% [6.1–10.0], FBS 7.1% [6.1–10.5] vs. 6.5% [5.0–9.5], RPMI 7.3% [7.1–11.6] vs. 7.2% [4.8–9.8]) and better resembled the amounts before cryopreservation (7.1% [6.4–8.1]). The relative amount of NK cells was significantly reduced further in SF (0.6% [0.5–0.9] vs. 0.7% [0.5–1.2])-stored MNCs and was trend-wise reduced in RPMI (0.5% [0.4–0.9] vs. 0.8% [0.4–1.2])-stored MNCs. For monocytes and T cells, we did not observe any significant differences comparing a resting period of 1 h to no resting (Figure 4A). Although the amount of total monocytes did not differ between unrested or rested MNCs, we found significant changes in the relative amounts of the individual subsets for CS, SF, and FBS. The relative proportion of classical monocytes was further significantly increased compared to unrested conditions (CS 80.3% [78.1–81.8] vs. 80.2% [78.5–85.2], SF 80.8% [78.5–82.8] vs. 86.2% [83.0–87.6], FBS 83.4% [80.9–84.9] vs. 85.4% [80.2–88.0], RPMI 82.3% [81.3–84.7] vs. 82.8% [80.7–87.6]), and the amount of the intermediate (CS 4.7% [3.6–5.6] vs. 3.5% [2.9–4.8], SF 4.1% [2.8–4.9] vs. 3.0% [2.6–4.1], FBS 4.5% [3.1–5.1] vs. 3.3% [2.4–4.7], RPMI 4.2% [3.1–5.0] vs. 3.5% [2.6–4.7]) and non-classical monocyte (CS 15.6% [12.5–18.0] vs. 14.9% [11.8–17.0], SF 15.4% [13.1–17.7] vs. 10.7% [9.1–14.3], FBS 12.2% [11.1–14.6] vs. 12.1% [9.4–14.5], RPMI 13.4% [11.7–15.8] vs. 13.1% [10.5–15.7]) subsets was further reduced significantly (Figure 4B). These changes were not observed for MNCs stored in RPMI. In summary, a 1-h resting period at 37°C in an incubator led to significant changes only in the amount of B cells and the relative distribution of the individual monocyte subsets in our experiments.

### 2.5. Changes in the Immunological Response to Bacterial Lipopolysaccharide after Storage in Different Cryopreservation Media

**Figure 5:**
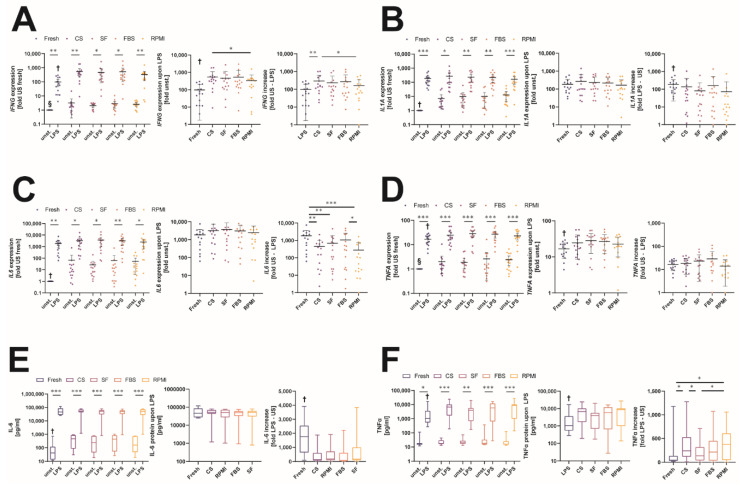
Changes in the immunological response to bacterial lipopolysaccharide after storage in different cryopreservation media. (**A**–**D**) In the respective left panels, expression of the cytokines *IFNG* (**A**), *IL1A* (**B**), *IL6* (**C**), and *TNFA* (**D**) is given as fold values relative to the individual fresh unstimulated amounts for all conditions and cryopreservation media. In the respective right panels, the increases upon LPS stimulation are shown for fresh samples and all cryopreservation media. (**E**,**F**) In the respective left panels, protein amounts of the cytokines IL-6 (E) and TNF-α (F) are given as pg/mL for all conditions and cryopreservation media. In the respective right panels, the increases upon LPS stimulation are shown for fresh samples and all cryopreservation media. * *p* < 0.05; ** *p* < 0.01; *** *p* < 0.001; † = significantly different from all other conditions; § = significantly different from other conditions except the ones indicated with “ns”. (*n* = 15 individual human donors).

Cryopreservation does not only affect the survival and relative distribution of different leukocyte populations, it can also interfere with the cells’ ability to respond to different inflammatory stimuli needed to fulfil their role in clearing altered or infected cells such as in modern chimeric antigen receptor (CAR) T cell therapy [27]. Therefore, we evaluated the effects of cryopreservation of MNC samples on the expression of different inflammatory cytokines and compared the alterations to samples before freeze–thawing. LPS was used to activate cellular inflammatory pathways in TLR4-expressing cells, evaluating the effects of different cryopreservation media on the reactivity of these MNCs. First, we measured mRNA levels of the four inflammatory cytokines interferon γ (*IFNG),* interleukin-1α (*IL1A*)*,* interleukin-6 (*IL6),* and tumor-necrosis factor α (*TNFA)*. MNCs showed an increase in the unstimulated transcription of *IFNG* after cryopreservation compared to the fresh condition (CS 7.4× [3.4–28.7], SF 7.0× [3.1–29.9], FBS 7.5× [1.1–25.5], RPMI 16.1× [4.1–35.8]). When stimulated using LPS, the expression of *IFNG* was significantly increased in all cryopreserved samples compared to fresh LPS-stimulated MNCs (Fresh 57.9× [12.2–201.9], CS 377.0× [230.2–565.6], SF 250.0× [109.2–447.4], FBS 267.4× [131.3–637.0], RPMI 321.2× [129.9–400.8]). By calculating the fold increases between unstimulated and LPS-stimulated MNCs for each condition, we found that the induction effect of *IFNG* mRNA on LPS was significantly higher in samples stored in CS (146.5× [82.7–460.3]) compared to fresh conditions (Fresh 57.9× [12.2–201.9]). This increased induction was not observed under the other freezing conditions (SF 139.8× [42.7–249.7], FBS 127.4× [43.6–323.5], RPMI 80.9× [31.4–199.8]) (Figure 5A).

For *IL1A,* we also observed a significantly increased basal production after cryopreservation compared to fresh levels (CS 3.0× [1.7–6.1], SF 5.1× [2.1–12.9], FBS 5.0× [1.4–9.3], RPMI 10.7× [2.5–14.6]). LPS stimulation increased the expression of *IL1A* in all samples, but we did not observe any significant difference between the fresh and cryopreserved MNCs (Fresh 122.8× [62.7–233.9], CS 100.9× [43.9–439.6], SF 105.9× [57.8–464.7], FBS 145.3× [57.0–360.8], RPMI 99.6× [48.2–280.4]). This resulted in significantly reduced fold increases upon LPS stimulation after cryopreservation compared to fresh conditions (Fresh 122.8× [62.7–233.9], CS 50.0× [4.8–111.5], SF 45.9× [6.2–93.5], FBS 59.2× [5.7–106.7], RPMI 9.7× [3.7–41.7]) (Figure 5B). Changes in *IL6* transcription were quite similar to those of *IL1A*. Cryopreservation led to an increased basal expression of the *IL6* transcript compared to fresh unstimulated MNCs (CS 7.4× [3.4–28.7], SF 7.0× [3.1–29.9], FBS 7.5× [1.1–25.5], RPMI 16.1× [4.1–35.8]). Upon LPS stimulation, the transcription of *IL6* mRNA was increased significantly (Fresh 1355× [274.1–2744], CS 2076× [564.2–4181], SF 2198× [371.0–4011], FBS 2346× [371.0–4011], RPMI 1073× [342.6–3634]). Again, there were no differences between fresh and cryopreserved samples, resulting in significantly reduced fold increases after thawing (Fresh 1355× [274.1–2744], CS 338.9× [22.1–656.6], SF 115.5× [22.7–807.3], FBS 294.1× [22.2–2227], RPMI 44.9× [16.3–573.2]). Only for FBS stored MNCs was the fold increase in *IL6* transcription not significantly different from that of fresh samples (Figure 5C). Further, cryopreservation and thawing significantly increased the basal, unstimulated expression of *TNFA* mRNA (CS 1.4× [0.9–1.7], SF 1.5× [0.8–2.2], FBS 1.2× [0.7–3.4], RPMI 1.9× [0.9–4.3]). When the MNCs were stimulated using LPS, *TNFA* expression was significantly increased in cells of all conditions compared to unstimulated ones. MNCs that were cryopreserved expressed significantly higher amounts of *TNFA* upon LPS stimulation compared to fresh ones (Fresh 17.1× [10.3–22.4], CS 19.8× [11.7–36.0], SF 26.2× [19.1–38.9], FBS 30.3× [14.8–33.6], RPMI 19.7× [13.33–35.3]). As the increased amounts of basal *TNFA* expression were accompanied by significantly increased levels of transcription upon LPS stimulation, fold increases in *TNFA* production before and after freeze–thawing (Fresh 17.1× [10.3–22.4], CS 17.2× [7.0–28.4], SF 23.6× [6.2–28.4], FBS 18.6× [8.8–40.0], RPMI 9.7× [5.4–20.3]) were not significantly different (Figure 5D).

We aimed to verify our findings on protein level after 24 h of stimulation. Basal IL-6 production was indeed increased after thawing in unstimulated MNCs (Fresh 42.4 pg/mL [13.6–119.2], CS 463.1 pg/mL [85.7–873.2], SF 237.2 pg/mL [45.7–756.1], FBS 442.1 pg/mL [52.8–848.0], RPMI 157.1 pg/mL [48.6–733.8]). LPS stimulation significantly increased the amount of IL-6 production compared to unstimulated conditions, but there was no significant difference between fresh and cryopreserved samples in terms of protein levels (Fresh 46,205 pg/mL [27,425–85,126], CS 52,348 pg/mL [43,616–70,031], SF 51,821 pg/mL [30,233–62,001], FBS 48,355 pg/mL [32,471–59,807], RPMI 50,022 pg/mL [27,512–71,611]). This resulted in reduced fold increases upon LPS stimulation for MNCs that were cryopreserved (Fresh 1750× [639.7–2541], CS 131.0× [70.7–612.4], SF 170.4× [79.3–991.9], FBS 80.8× [46.4–618.4], RPMI 182.7× [96.8–715.9]) (Figure 5E). Although *TNFA* expression was higher after thawing at a transcriptional level, basal TNF-α protein production was not significantly increased. LPS stimulation led to a significant increased production of TNF-α under all conditions and further, cryopreserved MNCs produced significantly higher amounts compared to fresh ones (Fresh 1049 pg/mL [543.2–3627], CS 6674 pg/mL [1694–10,200], SF 3977 pg/mL [690.5–5680], FBS 5754 pg/mL [644.4–12,060], RPMI 8690 pg/mL [994.3–10,311]) (Figure 5F). Thus, although MNCs preserved their ability to react to inflammatory stimuli after freeze–thawing, cryopreservation of MNCs can affect the inflammatory readouts observed. This impacts the usage of MNCs for functional assessment after cryopreservation, as the cellular composition and different pathways and cellular functions seem to be affected unequally.

### 2.6. Cryopreservation Affects the Activatability of the CD11b Surface Integrin

Bacterial LPS also induces the expression of CD11b in myeloid immune cells, leading to increased adhesion and further boosting TLR4 induced LPS signaling via a positive feedback loop in microbial infections [28,29]. Stimulation of monocytes in vitro using LPS alters the relative amount of different monocyte subsets by inducing shedding of membrane receptors used to identify CD16+ monocytes through A disintegrin and metalloproteinase 17 (ADAM17) [30]. Comparing the relative distribution of the individual monocyte subsets between unstimulated and LPS-stimulated samples under fresh and cryopreserved conditions in our study, we found that the relative amount of non-classical CD16^+^CCR2^−^ monocytes was diminished significantly, whereas the fraction of classical monocytes was increased significantly (Figure S4A).

**Figure 6:**
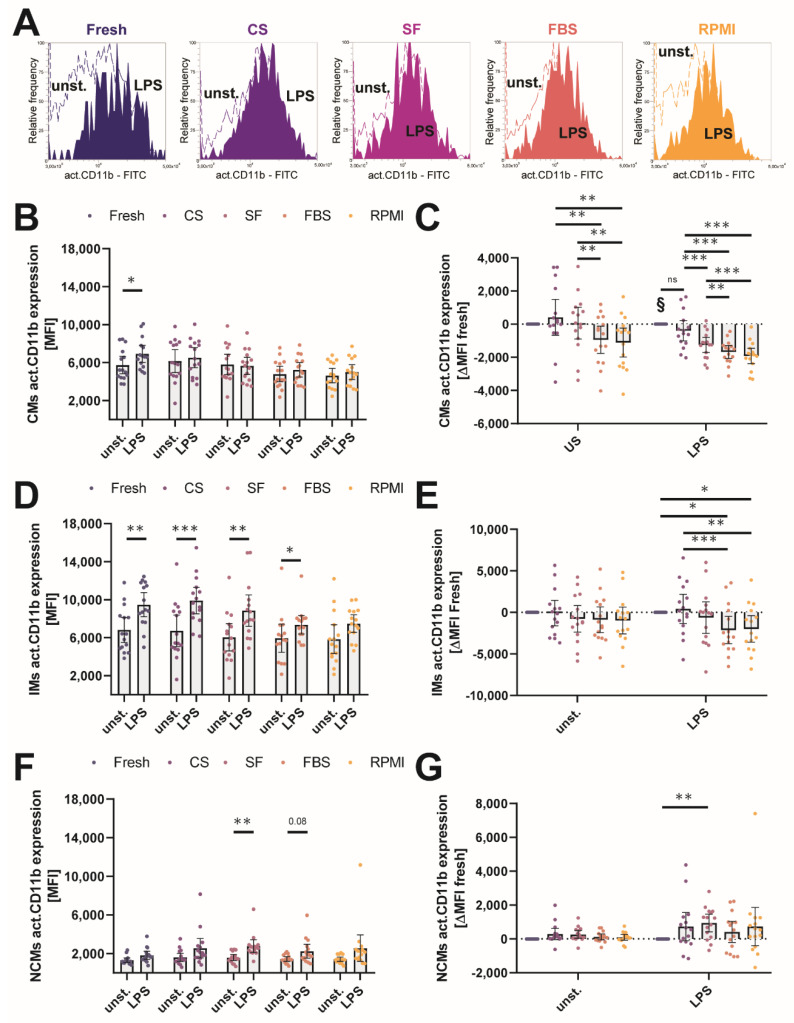
Cryopreservation affects the activatability of the CD11b surface integrin. (**A**) Representative flow cytometry overlays of the activated CD11b fluorescence of the gated intermediate monocyte subset for the fresh condition and cryopreservation media. Dotted lines represent the unstimulated monocytes, whereas filled lines represent the LPS-stimulated monocytes. (**B**) Median fluorescence intensity (MFI) of activated CD11b expression of the classical monocyte subset measured by flow cytometry for unstimulated and LPS-stimulated monocytes. (**C**) Delta fresh MFI values of cryopreserved classical monocytes either unstimulated or stimulated with LPS. Negative values indicate lower MFI compared to the individual fresh condition, and positive values indicate higher MFI compared to the individual fresh condition. (**D**) Median fluorescence intensity (MFI) of activated CD11b expression of the intermediate monocyte subset measured by flow cytometry for unstimulated and LPS-stimulated monocytes. (**E**) Delta fresh MFI values of cryopreserved intermediate monocytes either unstimulated or stimulated with LPS. Negative values indicate lower MFI compared to the individual fresh condition, and positive values indicate higher MFI compared to the individual fresh condition. (**F**) Median fluorescence intensity (MFI) of activated CD11b expression of the non-classical monocyte subset measured by flow cytometry for unstimulated and LPS-stimulated monocytes. (**G**) Delta fresh MFI values of cryopreserved non-classical monocytes either unstimulated or stimulated with LPS. Negative values indicate lower MFI compared to the individual fresh condition, and positive values indicate higher MFI compared to the individual fresh condition. * *p* < 0.05; ** *p* < 0.01; *** *p* < 0.001; § = significantly different from other conditions except the ones indicated with “ns”. (*n* = 15 individual human donors).

Previously, it has been shown that the individual monocyte subsets express different amounts of activated CD11b and that LPS mainly induces the expression of CD11b in intermediate monocytes [31]. We evaluated the effect of different cryopreservation media on the activatability of the surface integrin molecule CD11b by flow cytometry. MNCs were stimulated for 4 h with 100 ng/mL LPS, and the CD11b expression was analyzed by measuring the median fluorescence intensity in unstimulated/stimulated monocyte subsets before and after cryopreservation (Figure 6A). Similar to previous published data, CD11b expression was highest in the intermediate monocyte subset in our experiments, and in vitro stimulation with LPS led to an increase in CD11b expression in intermediate monocytes, further increasing the difference in CD11b expression between the classical and intermediate subsets (Figure S4B). In the classical monocyte subset, LPS stimulation only increased activated CD11b expression before cryopreservation (5205 MFI [4416–7160] vs. 6606 MFI [553.0–7796]), whereas we did not observe significant increases afterwards (Figure 6B). Overall, the absolute expression of activated CD11b in the classical monocyte subsets was significantly decreased after freeze–thawing in all cryopreservation media except CS (117 MFI [−528.0–2156]), with higher decreases in FBS (−578.0 MFI [−2308–411.0]) and RPMI (−2539 MFI [−2539–−100.0])-stored classical monocytes than in SF-stored ones (Figure 6C).

Interestingly, the LPS-induced increase in the CD11b expression of intermediate monocytes (Fresh 6399 MFI [4879–8300] vs. 8925 MFI [7654–11,801] was not significant in MNCs stored in RPMI (5308 MFI [3994–7353] vs. 7459 MFI [6544–8812]) and was markedly reduced in FBS (5785 MFI [3375–7328] vs. 7163 MFI [6365–8041])-stored MNCs (Figure 6D). When we compared the delta values of MFI for the unstimulated and LPS-stimulated groups, the FBS and RPMI group showed a significant decrease compared to the fresh condition and to CS-stored samples of LPS-treated MNCs (CS 169 MFI [-1614–3083], SF −51 MFI [−3594–867.0], FBS -2961 [−4381–231.0], and RPMI −2500 [−4395–201.0]) (Figure 6E). As expected, we did not observe large differences in the expression of active CD11b in the non-classical monocyte subset (Figure 6F). Non-classical monocytes cryopreserved in SF expressed significantly more active CD11b upon LPS stimulation compared to the other cryopreservation media and to the cells before freezing; fresh 557.0 MFI [−174.0–1293], CS 1231 MFI [102.0–1627], SF 525 MFI [−834.0–998.0], FBS 539 MFI [−459.0–1252]) (Figure 6G). Taken together, these observations show that the activatability of CD11b upon LPS stimulation was significantly reduced in MNCs cryopreserved in FBS or RPMI and also significantly altered in MNCs stored in SF, although the relative distribution of CD11b expression between individual monocyte subsets remained intact after cryopreservation.

### 2.7. Comparison of the Individual Cryopreservation Media for Different Experimental Research Questions

To give an overview of the performance of the individual cryopreservation media in this study, we created a table (Table S1) comparing the means and coefficients of variation between fresh and the individual cryopreservation media for all individual experiments. The media were ranked by their differences in the mean (deltas) and by their dispersion (coefficients of variation), allowing the selection of the appropriate cryopreservation medium for an individual research question (see Table 1).

## 3. Discussion

In clinical studies, the standardized collection, handling, cryopreservation, and thawing of MNCs has become a widely used and accepted method to systematically investigate the role of different immune cells in health and disease. The objective of this study was to compare the influence of four different cryopreservation media on the amount and function of MNCs, as there have been reports suggesting that cryopreservation can influence cell populations and their function [32,33].

The choice of cryopreservation medium affected the recovery and viability of MNCs after thawing in our study. The absolute recovery was higher when MNCs were stored in CS or FBS compared to samples frozen in SF or RPMI. This was paired with a higher viability of the recovered leukocytes, as shown by permeability assessment using Trypan blue or SytoxGreen. Although all media were sufficient to obtain live MNCs after thawing, the formulations of the individual media impacted the maximum gain. Similar observations were also found when others investigated changes in formulations for freeze–thawing of progenitor and stem cells [34,35,36].

Similar to prior published works [37,38], cryopreservation introduced quantitative changes in the distribution of different leukocyte populations in this study. In general, T cells were more likely to die during the cryopreservation process than other leukocyte populations, as the relative amount of CD3+ T cells of all recovered leukocytes was significantly reduced. In contrast, monocytes better withstood the procedure, as there was a relative overrepresentation of monocytes compared to all CD45+ cells after cryopreservation compared to the distribution before freezing. The unequal recovery rates between monocytes and T cells could be due to different susceptibilities of the individual leukocyte populations to the used cryoprotectants, as, for example, increasing dosages of DMSO led to significantly reduced recovery of Treg cells [39]. Interestingly, this increase was not evenly dispersed over all monocyte subsets, as the relative frequency of “younger” classical monocytes was increased and the amount of “aged” non-classical monocytes [40] was significantly reduced after cryopreservation. Although the respective changes were observed for all cryopreservation media, MNCs stored in CS showed the least significant changes among all cryopreservation media, and correlations of fresh and cryopreserved values were highest for this cryopreservation medium. These findings need to be considered in interpreting clinical studies investigating the association of circulating monocyte subsets in patients, as CD16+ monocytes were shown to correlate with disease severity and the occurrence of primary endpoints [41,42]. The unreflected usage of cryopreserved MNCs for such analyses could introduce biases, potentially leading to false associations. Overall, our findings suggest that only T cell analysis can be performed on frozen MNCs with an adequate representation of the initial distribution, as all other cell populations failed to show good correlations between cell numbers before and after freezing for each individual donor. It should be noted that due to the fact that for this study NK cells were defined as CD45+CD3-CD56+ and gating was done very restrictively on higher CD56 expression, the amount of NK cells in our study was somewhat lower than that observed in other studies that focused in more detail on NK cell subpopulations. Due to missing CD16 staining in the analysis panel, we were not able to study the CD45+CD3-CD16+CD56- NK cell population [43].

As a 1-h resting period led to a recovery of the relative distribution of CD14+ cells in the study of Lemieux et al., we also evaluated the effect of a 1-h resting period in full MNC medium on the relative distribution of different cell populations in our study [19]. However, we only observed a normalization of B cells to amounts before cryopreservation. In contrast, a resting time of 1 h in cell suspension further diminished the relative amount of intermediate and non-classical monocytes in our analysis.

It has also been stated that cryopreservation alters the capacity of MNCs to react to different stimuli when compared to fresh samples [16,27,44]. We therefore asked whether the choice of cryopreservation medium would affect the responsiveness of the MNCs to LPS and if one medium would perform better than another. We analyzed the expression and production of different cytokines at basal levels and upon LPS stimulation. LPS stimulation led to a significant increase in the production of inflammatory cytokines compared to basal values. Cryopreserved MNC samples showed an increased expression of all inflammatory cytokines compared to fresh samples. Fold-wise, this basal change ranged from a 3- up to a 100-fold change compared to fresh samples. As total *18S* RNA was used to normalize the quantification data and cytokine production was probably driven mainly by myeloid cell populations in this experiment, the altered monocyte–lymphocyte ratios observed after freeze–thawing have to be taken into account too with respect to biasing the observed basal change. These findings suggest that basal inflammatory activity could be overestimated in study cohorts using cryopreserved MNCs. When analyzing fold inductions, the response profiles of the individual cytokines need to be considered, as fold changes were different between the individual cytokines determined. Interestingly, when we evaluated the response of monocyte subsets to express activated CD11b on their surface upon LPS stimulation, we observed profound differences between the individual media. Monocytes stored in medium containing FBS (FBS and RPMI groups) showed diminished expression of activated CD11b, and the induction of CD11b upon LPS was significantly lower in FBS- and RPMI-stored MNCs, with only CS-stored cells being non-significantly changed compared to fresh samples.

To conclude, we report that choosing a particular cryopreservation medium can have dramatic impacts on the obtained readouts and can introduce alterations in the recovery, survival, and distribution of different immune cell populations after thawing. For all cell types studied, alterations induced by freeze–thawing need to be considered when different studies or experimental samples are compared. We summarize and rank the observed changes for different experimental setups and for individual cell populations in an overview table, aiding other researchers in selecting an appropriate cryopreservation medium.

## 4. Materials and Methods

### 4.1. Study Samples

Buffy coats were obtained from leukocyte reduction system chambers (LRSC) obtained by single platelet apheresis of 15 healthy individuals of both sexes. Due to technical reasons, LRSCs used to isolate buffy coats in this study were stored overnight at 4°C before processing. The advantage of leukapheresis compared to whole-blood samples is the large yield and fast isolation of mononuclear cells, allowing one to test for large-scale experimental research setups [45]. All donors gave their written informed consent before the leukocyte reduction chambers were used for MNC isolation. The study was performed after approval by and according to recommendations of the ethical committee of the Medical University of Vienna (EK 1575/2014).

### 4.2. MNC Isolation

MNCs were collected by density gradient centrifugation as published before [46]; please see supplemental information. In short, the content of the leukocyte reduction chambers was diluted with phosphate-buffered saline (PBS, Sigma–Aldrich, St. Louis, MO, USA); 15 mL of Lymphocyte Separation Medium 1077 (Promocell, Heidelberg, Germany) was added to the lower chamber of SepMate-50 Tubes (Stemcell, Vancouver, Canada), and the diluted content of the leukocyte reduction chambers was added on top of the inner barrier. The special insert of these tubes allows density gradient centrifugation at 1200 g for 10 min with full break, significantly reducing the processing time of MNC isolation. The buffy coat was washed once with PBS and resuspended at 2 × 10^7^ cells/mL in full MNC medium [RPMI1640 (Sigma–Aldrich, St. Louis, MO, USA) supplemented with 10% fetal bovine serum (FBS Gold; Seraglob, Switzerland), glutamine, and antibiotics (both Lonza, Basel, Switzerland)].

### 4.3. Cryopreservation

After an aliquot was taken to perform experiments on fresh MNCs, the remaining MNCs were split into four aliquots of 5 × 10^7^ cells each for the different cryopreservation media. Freezing was performed serially for each aliquot to ensure equal stay times as cell pellets and in cryopreservation medium before freezing. Four widely used cryopreservation media were assessed, two commercially available ones and two self-prepared ones: (1) CryoStor CS10 Cell Freezing Medium (Stemcell, Vancouver, Canada); (2) Synth-a-Freeze Cryopreservation Medium (ThermoFisher, Waltham, MA, USA); (3) 90% FBS Gold (Seraglob) + 10% dimethyl sulfoxide (DMSO, >99.9% purity, Sigma–Aldrich, St. Louis, MO, USA); (4) 70% RPMI1640 + 20% FBS Gold + 10% DMSO. FBS batch-to-batch comparability was assured by usage of FBS Gold, which is a defined fetal bovine serum composed of chromatographic isolated and re-supplemented components. The MNCs were centrifuged at 500× *g* for 5 min and resuspended on ice in the respective cryopreservation medium at a concentration of 2 × 10^7^ cells/mL. The cell suspension was split into five aliquots of 1 × 10^7^ cells each, transferred into 2 mL Bio-One Cryo.s Tubes (Greiner, Kremsmuenster, Austria) and placed at −80°C into a CoolCell reduced-scale freezing container (Corning, Corning, NY, USA), cooling the MNCs at a rate of −1°C/min. Afterwards, the cryo-tubes were transferred into liquid nitrogen tanks and stored there until thawing.

After 6–10 weeks, the MNCs were thawed again by placing the cryo-tubes at room temperature until the outer layer of the crystal became liquid. Then, the crystal containing the MNCs was flipped into ice-cold full MNC medium. After all of the cryopreserved medium became liquid, the MNCs were washed once and resuspended in 1 mL pre-warmed full MNC medium for the assessment of recovery and viability.

### 4.4. Measurement of Absolute Cell Counts and Viability after Thawing

Recovery and viability of MNCs after thawing were assessed using the LUNA Automated Cell Counter (Logos Biosystems, Anyang, South Korea) together with 0.4% Trypan Blue Solution (Lonza, Basel, Switzerland). The cell suspensions were adjusted to 2 × 10^7^ viable cells/mL, and the concentration was verified using a XN-350 Hematology Analyzer (Sysmex, Vienna, Austria).

### 4.5. In Vitro MNC Stimulation

Cell function prior to and after thawing was assessed by stimulation of MNCs with the toll-like receptor 4 agonist lipopolysaccharide (LPS, from *Escherichia coli* strain O111:B4, Sigma–Aldrich, St. Louis, MO, USA). MNCs were seeded at a concentration of 2 × 10^5^ cells/400 µL full MNC medium and were left unstimulated or were stimulated with 100 ng / mL LPS either for 4 h for all gene expression experiments or for 24 h to collect the condition culture supernatant. For experiments involving a 1-h resting period, cells in full MNC medium were incubated for 60 min under standard cell culture conditions (humidified atmosphere, 5% CO_2_, 37°C) before analysis and stimulation. After the indicated times, cells were harvested and conditioned culture medium was collected by taking the supernatant. Cell-free supernatant was generated by centrifugation at 500× *g* for 5 min.

### 4.6. Flow Cytometry

For flow cytometric quantification of leukocyte populations, three aliquots of 1 × 10^5^ MNCs were stained with different antibody panels (all antibodies were purchased from Biolegend, San Diego, CA, USA) in 10 µL PBS for 15 min at room temperature. The individual panels were as follows: (1) Monocyte panel: CD45 (Clone 2D1, Alexa Fluor 700), HLA-DR (Clone L243, APC/Fire750), CD14 (Clone HCD14, APC), CD16 (Clone 3G8, BrilliantViolet 605), CCR2 (Clone K036C2, Brilliant Violet 421); (2) T cell panel: CD45 (Clone 2D1, Alexa Fluor 700), CD3 (Clone OKT3, APC), CD4 (Clone A161A1, APC/Fire750), CD8 (Clone Hit8a, Brilliant Violet 421), CD28 (Clone CD28.2, Brilliant Violet 605); (3) B/NK cell panel: CD45 (Clone 2D1, Alexa Fluor 700), CD3 (Clone OKT3, APC), CD19 (Clone HIB19, Brilliant Violet 421), CD56 (Clone QA17A16, APC/Fire750). Afterwards, the cell suspensions were incubated for 5 min with 220 µL of PBS supplemented with 30 nM SytoxGreen viability dye (ThermoFisher, Waltham, MA, USA) and were analyzed immediately on an Attune NxT Flow Cytometer (ThermoFisher, Waltham, MA, USA). MNCs treated with LPS and their respective controls were incubated similarly as above using the Monocyte Antibody Panel, but instead of a viability dye, an antibody against activated CD11b (Clone CBRM1/5, FITC, Biolegend, San Diego, CA, USA) was used during incubation and the cell suspensions were washed using only PBS.

### 4.7. Gene Expression Analysis

RNA from MNC lysates was prepared using Maxwell RSC simplyRNA Tissue kits on a Maxwell RSC Instrument (both Promega, Madison, WI, USA). To create cDNA from the extracted RNA, 10 μL RNA was added to 10 μL GoScript 1:1 Oligo(dT)/Random Primer Reverse Transcriptase Mix (Promega, Madison, WI, USA), and reverse transcription was performed on a Thermocycler (Analytika Jena, Jena, Germany). To quantify the expression of different mRNAs, qPCR was performed on a CFX Connect Real-Time PCR Detection System (BioRad, Hercules, CA, USA). For the detection of different genes, primers were designed using the Universal Probe Library (UPL) system (Roche, Basel, Switzerland), and qPCR was performed using GoTaq Probe qPCR Master Mix (Promega, Madison, WI, USA). Primer sequences and used UPL probes were as follows: *18S* (FWD-gggttcgattccggagag, REV-tcgggagtgggtaatttgc, UPL 40), *IFNA* (FWD-ggcattttgaagaattggaaag, REV-tttggatgctctggtcatctt, UPL 21), *IL1A* (FWD-ggttgagtttaagccaatcca, REV-tgctgacctaggcttgatga, UPL 6), *IL6* (FWD-gatgagtacaaaagtcctgatcca, REV-ctgcagccactggttctgt, UPL 40), *TNFA* (FWD-cagcctcttctccttcctgat, REV-gccagagggctgattagaga, UPL 29). Ct values were calculated by regression and were normalized by the ΔCt method relative to 18S as a housekeeping gene.

### 4.8. Protein Quantification

Cytokine production was analyzed in the supernatant of MNCs before and after cryopreservation. Reactiveness of the immune response to a bacterial stimulus was assessed by stimulating the cells with LPS as stated above. Secreted protein amounts were quantified using the TNF alpha Human Uncoated ELISA Kit with plates as well as the IL-6 Human Uncoated ELISA Kit (both ThermoFisher, Waltham, MA, USA) as indicated by the manufacturers’ instructions.

### 4.9. Statistical Analysis

Statistical analysis was performed using Prism 8 for Windows (Graphpad Software, San Diego, CA, USA). Unless otherwise stated, the effects of cryopreservation media were assessed using RM (paired) one-way ANOVA, followed by a post-hoc analysis to account for multiple testing. Normality was assessed before, using the D’Agostino–Pearson normality test. Dunnett’s post-hoc test was used to compare the samples after cryopreservation to conditions before freezing. Afterwards, the means between different media were compared against each other, and adjustment for multiple comparison was performed using Tukey’s post-hoc test. To reliably compare dispersion of the measured values under the individual conditions, coefficient of variation (standard deviation normalized relative to the mean) values were used. Unless otherwise stated, summary data are shown as bar charts with mean ± s.d. and all data points. In the main text results, the detailed data for all experiments are given in round brackets with median values with unit and inter-quartile range in the form of the 25th to 75th percentile in square brackets (MEDIAN UNIT [IQR]).

## Figures and Tables

**Table 1:** Summarized ranking of the individual cryopreservation media. Cumulative ranks of the geometric mean values of the ranks in the individual experimental research questions. Best rank = 1, worst rank = 4. The individual rankings for each subpanel are given in the Table S1.

Experimental Research Question	CS	SF	FBS	RPMI
Recovery and Viability (Figure 1)	2	4	1	3
Cell population relative amount (Figure 2)	1	3	2	4
Cell population dead cells (Figure 2)	1	4	2	3
Monocyte subsets (Figure 3)	1	2	4	3
T cell subsets (Figure 3)	4	1	2	2
Cytokine measurement (Figure 5)	2	1	4	3
Monocyte CD11b activation (Figure 6)	1	3	2	4
Cumulative performance (overall rank)	1	3	2	4

## Data Availability

All data from this study are available from the corresponding author upon request.

## Supplementary Materials

The following supporting information can be downloaded at:www.mdpi.com/article/10.3390/ijms23031881/s1.
