# Supplementary material for: Quantitative and Functional Assessment of the Influence of Routinely Used Cryopreservation Media on Mononuclear Leukocytes for Medical Research
Source: Int J Mol Sci. 2022 Feb 7;23(3):1881. doi: 10.3390/ijms23031881 (PMC8837123; doi:10.3390/ijms23031881)
Supplement: Supplementary file 1 [file ijms-23-01881-s001.zip › ijms-1550965-supplementary.pdf]

Supplementary Material

for

Quantitative and Functional Assessment of the Influence of Routinely Used Cryopreservation Media on Mononuclear Leukocytes for Medical Research

Patrick Haider[1,2], Timothy Hoberstorfer[1], Manuel Salzmann[1], Michael B. Fischer[3], Walter S. Speidl[1,2], Johann Wojta[1,2,4], Philipp J. Hohensinner[2,5]

[1]Department of Internal Medicine II/Division of Cardiology; Medical University of Vienna; Vienna; Austria

[2]Ludwig Boltzmann Institute for Cardiovascular Research; Medical University of Vienna; Vienna; Austria

[3]Department of Blood Group Serology and Transfusion Medicine; Medical University of Vienna; Vienna; Austria

[4]Core Facilities; Medical University of Vienna; Vienna; Austria

[5]Center for Biomedical Research; Medical University of Vienna; Vienna; Austria

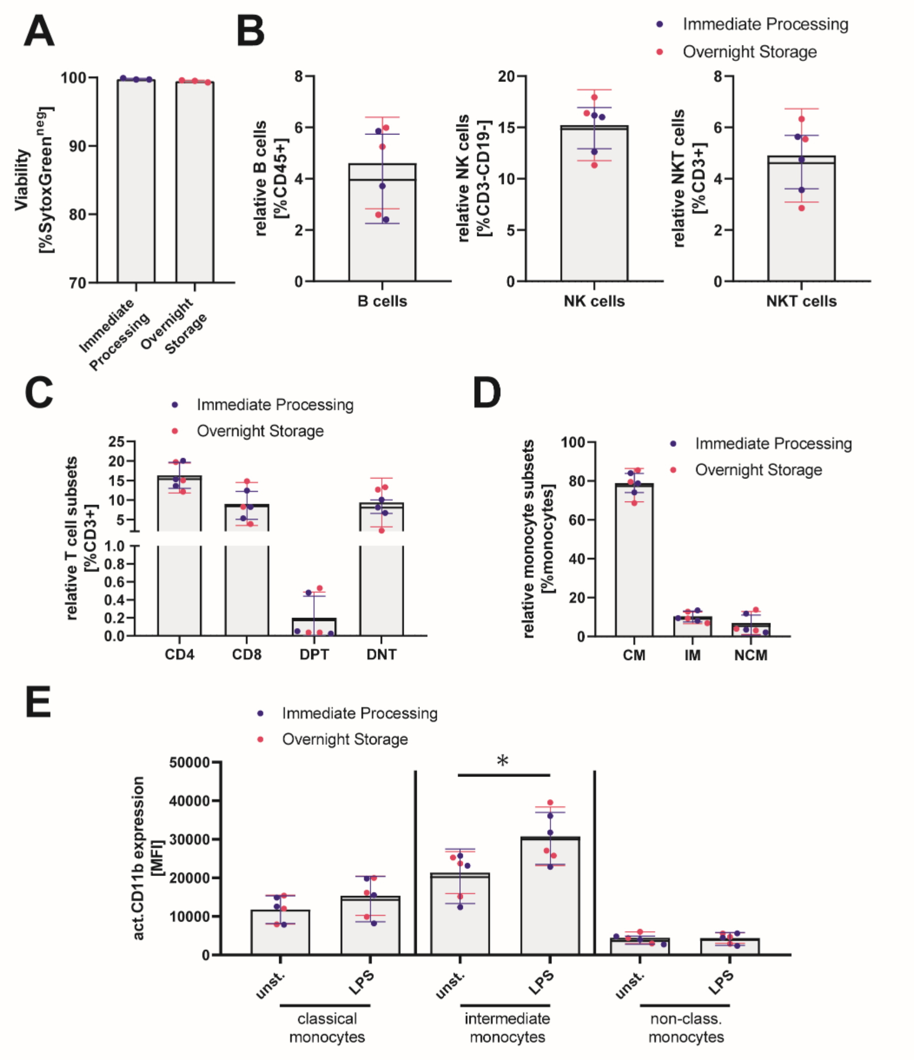

Figure S1: Comparison of immediately processed vs. overnight stored LRSCs. The influence of overnight storage of LRSC at 4°C was assessed in a validation cohort to assess if storage induces a bias compared to immediate processing after leukapheresis. (**A**) Viability assessment by SytoxGreen nuclear staining. (B) Relative amounts of B cells, NK cells and NKT cells. (C) Relative amounts of T cell subpopulations (CD4, CD8, DPT, DNT). (D) Relative distribution of monocyte subsets (CM, IM, NCM). (E) MFI of activated CD11b expression on different monocyte subsets which were left unstimulated or were stimulated with 100 ng/ml LPS. * indicates $p < 0.05$ between unstimulated and LPS stimulated intermediate monocytes. * $p < 0.05$ (n = 3 individual human donors).

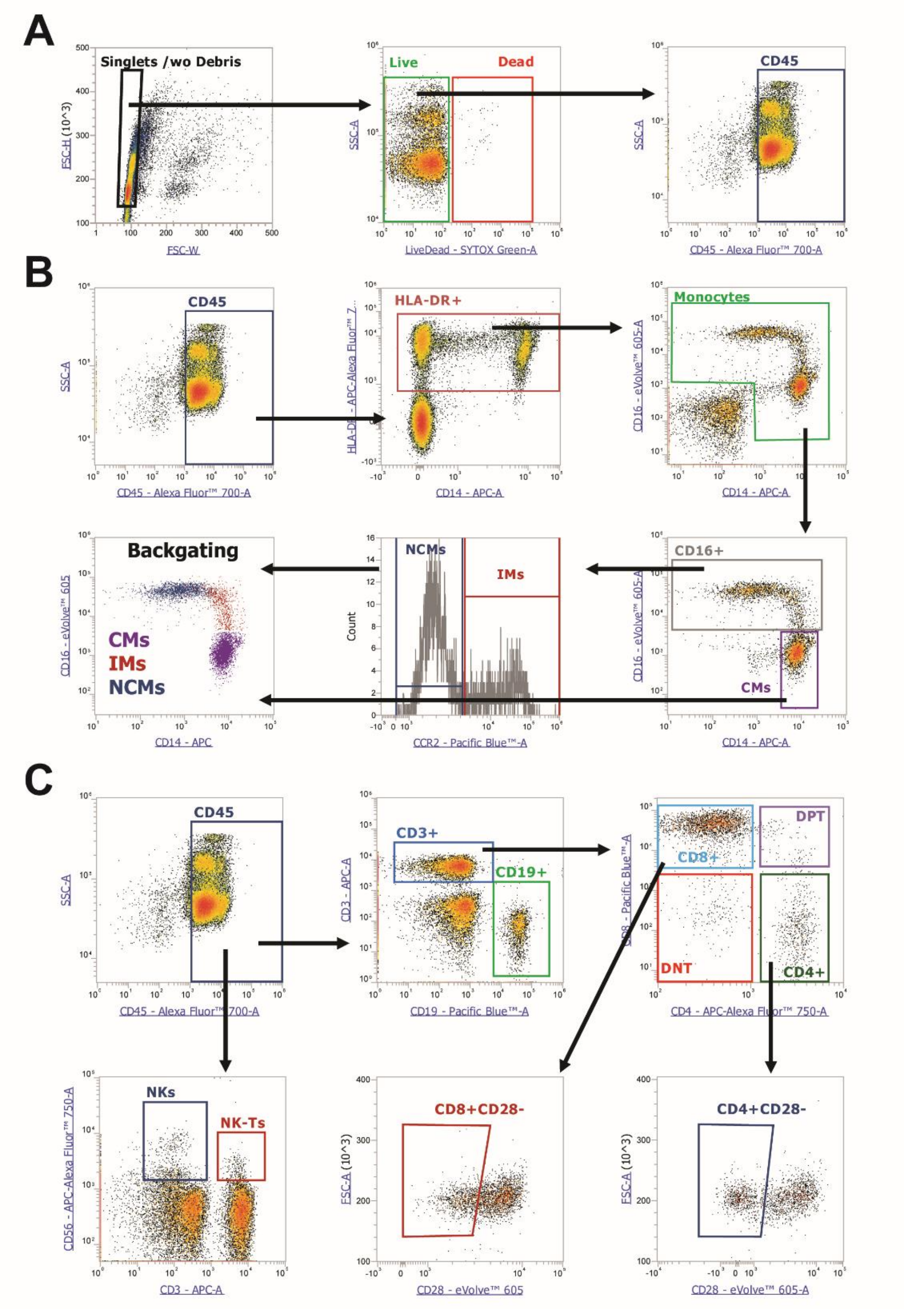

Figure S2. Flow cytometric gating scheme. (**A**) Gating strategy for singlets, live/dead cells and CD45+ leukocytes. (**B**) Gating strategy for monocytes and their subsets. The cells were pre-gated according to Panel A. (**C**) Gating strategy for lymphocyte subsets. The cells were pre-gated according to Panel A.

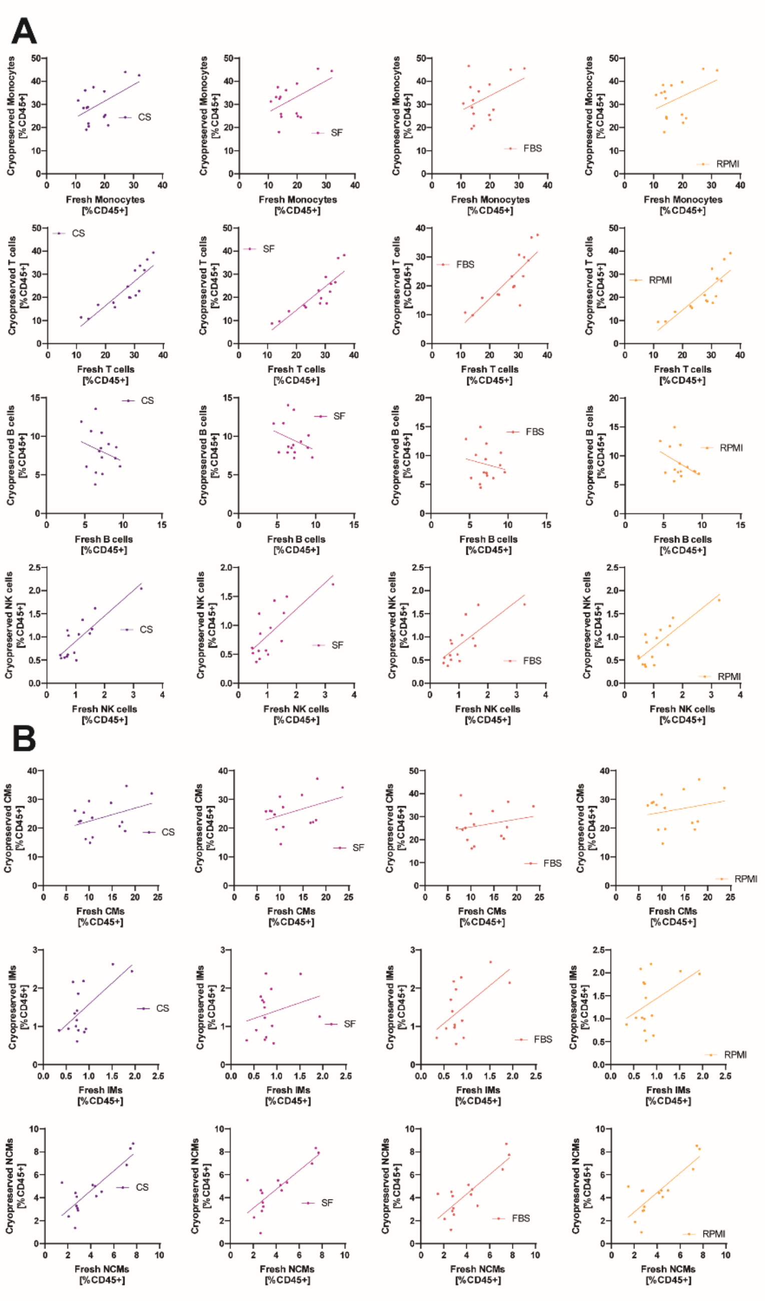

Figure S3. : Individual correlation plots between fresh and cryopreserved samples for all MNC subpopulations. (**A**) Correlation plots of relative cell amounts of monocytes, T cells, B cells and NK cells before and after cryopreservation for each individual cryopreservation medium and cell population. Correlation was analyzed by linear regression. (B) Correlation plots of relative monocyte subset distribution before and after cryopreservation for each individual cryopreservation medium and cell population. Correlation was analyzed by linear regression.

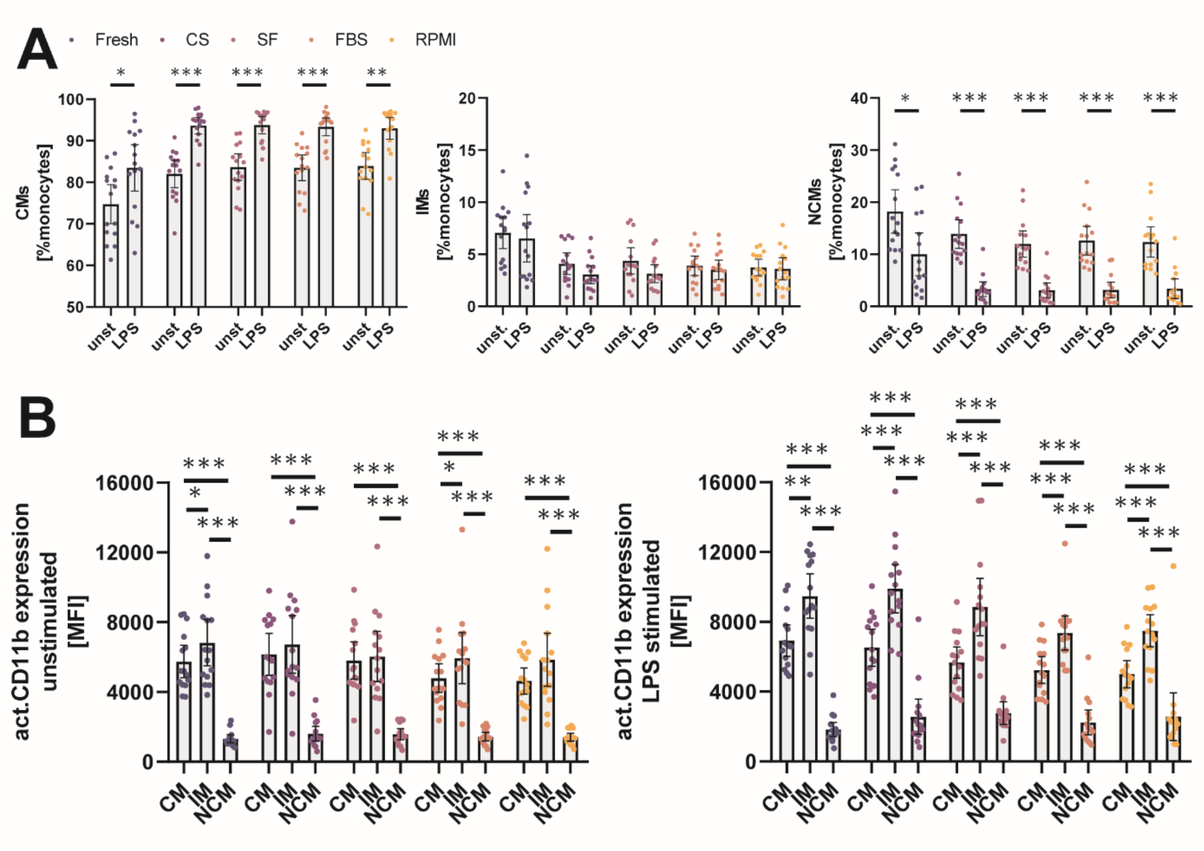

Figure S4. Cryopreservation affects the activatability of the CD11b surface integrin. (**A**) Relative distribution of the different monocyte subsets measured by flow cytometry for unstimulated (left respective bar) and LPS stimulated (right respective bar) cells. (**B**) Median fluorescence intensity (MFI) of activated CD11b expression of the different monocyte subsets measured by flow cytometry for unstimulated (left panel) and LPS stimulated (right panel) cells. CM = classical monocytes, IM = intermediate monocytes, NCM = non-classical monocytes. * $p < 0.05$; ** $p < 0.01$; *** $p < 0.001$. ($n = 15$ individual human donors).
